# Enhancing inpatient psychotherapeutic treatment with online self-help: study protocol for a randomized controlled trial

**DOI:** 10.1186/s13063-015-0620-6

**Published:** 2015-03-17

**Authors:** Rüdiger Zwerenz, Jan Becker, Rudolf J Knickenberg, Karin Hagen, Michael Dreier, Klaus Wölfling, Manfred E Beutel

**Affiliations:** Department of Psychosomatic Medicine and Psychotherapy, University Medical Center, Johannes Gutenberg-University, Untere Zahlbacher Str. 8, 55131 Mainz, Germany; Clinic for Psychosomatic Rehabilitation, Rhön-Klinikum AG, Salzburger Leite 1, 97616 Bad, Neustadt/Saale, Germany

**Keywords:** Depression, Psychosomatic inpatient treatment, Online self-help, Randomized controlled trial

## Abstract

**Background:**

Depression is one of the most debilitating and costly mental disorders. There is increasing evidence for the efficacy of online self-help in alleviating depression. Knowledge regarding the options of combining online self-help with inpatient psychotherapy is still limited. Therefore, we plan to evaluate an evidence-based self-help program (deprexis®; Gaia AG, Hamburg, Germany) to improve the efficacy of inpatient psychotherapy and to maintain treatment effects in the aftercare period.

**Methods/design:**

Depressed patients (n = 240) with private internet access aged between 18 and 65 are recruited during psychosomatic inpatient treatment. Participants are randomized to an intervention or control group at the beginning of inpatient treatment. The intervention group (n = 120) is offered an online self-help program with 12 weekly tasks, beginning during the inpatient treatment. The control group (n = 120) obtains access to an online platform with weekly updated information on depression for the same duration. Assessments are conducted at the beginning (T0) and the end of inpatient treatment (T1), at the end of intervention (T2) and 6 months after randomization (T3). The primary outcome is the depression score measured by the Beck Depression Inventory-II at T2. Secondary outcome measures include anxiety, self-esteem, quality of life, dysfunctional cognitions and work ability.

**Discussion:**

We expect the intervention group to benefit from additional online self-help during inpatient psychotherapy and to maintain the benefits during follow-up. This could be an important approach to develop future concepts of inpatient psychotherapy.

**Trial registration:**

ClinicalTrials.gov Identifier: NCT02196896 (registered on 16 July 2014).

## Background

Depression poses one of the most serious health problems on a worldwide basis [1]. While effective evidence-based treatments have been established, only a minority of patients receive them. Thus, there have been many efforts to deliver online interventions, which have the potential to be widely available. Nevertheless, it is still unclear if and how people use the internet if they have mental health problems. In a recent survey in the German population, Eichenberg and colleagues [2] found that more than one quarter (26.3% of the general population resp. 43.7% of the internet users) consider seeking help online, but only a few (2.2%) already used psychological counseling or could imagine (<10%) seeking support online in case of psychiatric strain. Indeed, positive effects of internet-based interventions on symptoms have been established regarding depression. In two recent meta analyses effect sizes have been reported, with small effects for the difference between self-guided psychological treatment of depression and control groups (d = 0.28, seven randomized controlled trials [3]) and moderate effects (d = 0.56; 19 studies [4]) for the post-treatment effects. For anxiety disorders another meta-analysis found an overall effect size of computer-aided psychotherapy compared with non-computer-aided psychotherapy of d = 1.08 (23 randomized controlled trials [5]). A considerable number of studies and meta-analyses have found that guided self-help for depression is effective compared to untreated control conditions [3] and that it may be as effective as face-to-face treatments [5,6].

Most research has focused on guided online self-help interventions, including support by a professional therapist or coach. However, as determined by a meta-analysis [4], supported interventions increased retention compared to studies without support, and this leads to the recommendation of blended approaches in the internet-based treatment of depression. Johansson and Andersson [7] also found a strong correlation between the degree of therapist support and outcome of internet-based psychological treatments of depression. Evidence regarding the effectiveness of online-based treatments as a complement to regular medical or psychotherapeutic care, however, is limited and contradictory [7].

deprexis® is a new and interactive internet-based self-help program that does not require therapist support. It has found strong support in the treatment of depressive disorders [7-9]. However, its efficacy has not yet been investigated as an adjunct to inpatient psychotherapy. We have only found one published study protocol in which it is planned to use deprexis® as an adjunct to psychological treatment in various settings [10].

Depression is the most frequent diagnosis in inpatient psychotherapy [11], which is indicated when depression is severe, significantly impairing activities of daily living and work ability or if it is compounded by other mental disorders [12]. Depression can be improved considerably by inpatient psychotherapy [13,14]. However, despite intensive treatment, many patients do not fully remit in a time frame of 4 to 8 weeks. Partially remitted or subclinical depression is known to be a risk for relapse [15], particularly when no immediate follow-up outpatient treatment is provided. Indeed, following discharge, a substantial proportion of patients need to search for a psychotherapist, and have to wait for adequate treatment for several months. An internet-based treatment could bridge this gap and offer immediate support after inpatient treatment. A large randomized controlled trial (n = 400 patients) could show that treatment effects were maintained until a 12-month follow-up after inpatient psychosomatic treatment with a 12-week transdiagnostic internet-based maintenance treatment compared to treatment as usual [16]. Internet-based interventions are further effective in preventing relapse; for example, with internet chat groups following inpatient psychotherapy [17] or a 10-week internet-based cognitive behavior therapy compared to a control group [18].

We assume that adding an online self-help program to standard inpatient treatment could increase the efficacy of inpatient treatment and also bridge the gap to outpatient treatment. Therefore, this trial aims to determine the feasibility and effectiveness of supplementing online self-help for depressed patients during and immediately after inpatient psychotherapy.

## Methods

### Participants

At admission to inpatient psychotherapy, patients are informed about the study. Those eligible are inpatients aged 18 to 65 years who have private internet access, sufficient German language proficiency, a score in the clinical range (>13) of the Beck Depression Inventory-II (BDI-II) [19] and a clinical diagnosis of depression (International Statistical Classification of Diseases and Related Health Problems, 10th revision (ICD-10), codes F32.x, F33.x, F34.1, F43.2), as assessed by the individual therapist of the patient. Those excluded are patients suffering from psychosis, current alcohol or drug addictions, borderline, antisocial, schizoid and schizotypal personality disorders, anorexia nervosa, lifetime diagnoses of schizophrenia, schizoaffective, bipolar or organic mental disorder.

The study assistant screens every patient at intake who fulfils the BDI-II criteria in the standard intake assessment and who is diagnosed with a depressive disorder by his or her therapist regarding inclusion and exclusion criteria.

Data for patients who are eligible for participation and have given written informed consent are coded, and patients are randomized by the study center. Study information is given to the patients by a study assistant. They are provided with their access codes for the online self-help program or the online platform of the control condition and receive a short introduction to their respective treatment condition.

The Study Center of Mental Disorders at the University Medical Center of the Johannes Gutenberg-University Mainz is responsible for storing personal data and randomizing participants. Administration of the internet-platform and allocation of the weekly information text in the control condition are managed by psychologists of the Department of Psychosomatic Medicine and Psychotherapy of the University Medical Center Mainz.

The clinical protocol and written informed consent were approved by the Ethics Committee of the Federal State of Rhineland Palatinate (Germany), which is responsible for the coordinating center in Mainz (Ref. No. 837.093.14(9332-F)). All procedures described in the clinical trial protocol (ClinicalTrials.gov identifier: NCT02196896) follow the International Conference on Harmonisation Good Clinical Practice guidelines and the ethical principles described in the current revision of the Declaration of Helsinki. The trial will be carried out in keeping with local legal and regulatory requirements.

The study platform containing the information for the control group and redirecting all participants to the website used for the online survey is located on a firewall-protected webserver which uses an SSL-encrypted (secure sockets layer) access to the MySQL-database containing the login information. SSL-encryption is a security standard widely used on the internet (for example, for online banking). All questionnaires will be administered via the online survey program SoSci Survey (https://www.soscisurvey.de) that uses SSL-coded internet connections as well. Furthermore, all patients use pseudonyms to login to the study platform. As no personal data are stored on the webserver, identification of the real identity of the user is not possible.

### Intervention

In addition to inpatient treatment (treatment as usual) consisting of individual and group psychotherapy, integrating body-oriented and creative psychotherapy interventions, as well as various adjunct treatments (for example, relaxation, patient education, exercising, therapeutic community) [20], participants in the intervention group receive access to a 12-week internet-based self-help treatment (deprexis®) that is described in detail elsewhere [21]. The self-help program consists of 10 modules, plus one introductory and one summary module. The modules combine cognitive and behavioral techniques with positive psychology, dream work and emotion-focused interventions. The contents of the modules are provided as a simulated dialogue, explaining and illustrating concepts and techniques, engaging the user in exercises, and continuously asking for user feedback. Provision of subsequent content is tailored to the users’ responses. Participants can work through the program at their own pace, as modules are not gradually made available at a specific schedule [10]. If desired by the user, the program automatically delivers helpful comments or reminders on a daily basis by SMS or email.

In the weekly treatment plan delivered to the patients by the clinic, two time slots are scheduled for the intervention per week on different weekdays. Participants work in a room with computer terminals at their own pace during these time slots. After discharge from the hospital, patients are eligible to continue the online self-help program free of charge for the remaining period (the program is accessible for a total of 12 weeks from first login).

### Control condition

All participants receive inpatient treatment (treatment as usual) as described above. Participants in the control condition additionally receive access to an online platform with freely available and reliable information regarding the diagnosis, etiology, course and treatment of depression, including self-help options. Information has been compiled with appropriate permissions from public domain sources, for example the patient version of the German medical guidelines or information provided by the German network on depression, health insurance companies or federal health agencies. In order to increase motivation and ease of studying, information is broken down into packages with specific topics, which are made accessible on a weekly basis. In order to ensure comparability to deprexis®, two time slots with the same duration are allotted for program participation during inpatient treatment. Participants may use the webpage over a period of 12 weeks.

### Assessment

**Figure 1:**
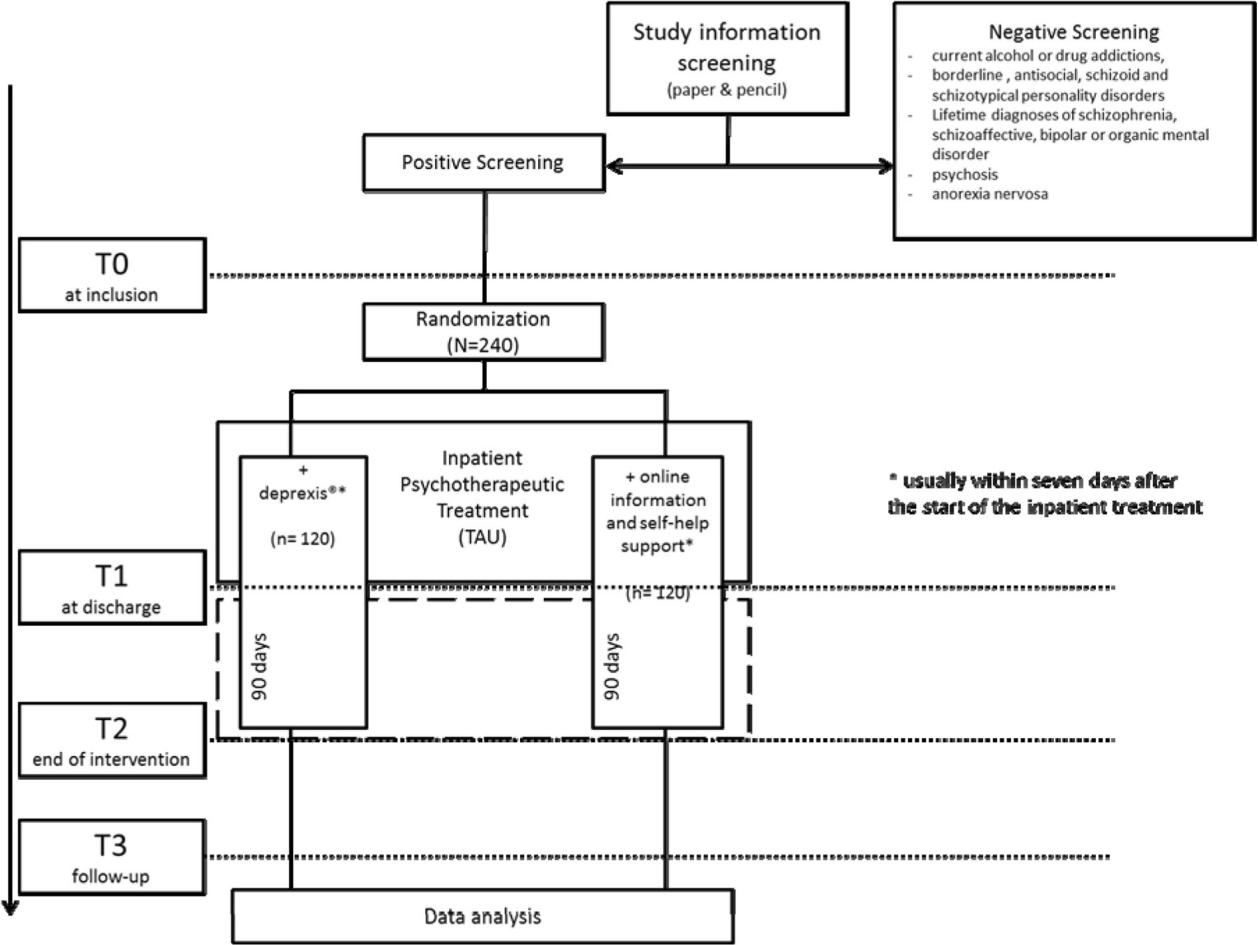
**Study design and time points of assessment.** TAU, treatment as usual.

Assessments are conducted at the beginning (T0) and the end (T1) of inpatient treatment, at the end (T2) of intervention and 6 months after randomization (T3; Figure 1). Screening is based on the BDI-II [19], a reliable and valid 21-item self-report scale of depression, during the past 2 weeks, together with ICD-10 diagnoses made by the individual therapist. At T0 patient characteristics (for example, education, employment, family status) will be taken from the basic documentation of the clinic. In addition to the BDI-II, the reliable and valid Patient Health Questionnaire (PHQ-9) is used to assess depression [22] along with the Generalized Anxiety Screener (GAD-7) [23]. Other self-report questionnaires assess self-esteem (Rosenberg Self-Esteem Scale, RSE) [24], quality of life (European Health Interview Survey Quality of Life 8 Item Index, EUROHIS-QOL 8) [25] and dysfunctional depression-related cognitions (Dysfunctional Attitude Scale, DAS) [26]. We further assess childhood trauma with the Childhood Trauma Questionnaire (CTQ) [27]. The Work Ability Index (WAI) [28] questions current work ability in relation to physical and mental job demands, and prognosis for the forthcoming 2 years. Structural psychological deficits are assessed by the short form of the OPD Structure Questionnaire (OPD-SQ) [29], and the therapeutic alliance by the Helping Alliance Questionnaire (HAQ) [30], with a patient as well as a therapist rating.

At discharge (T1) and follow-up (T3) subjects fill out the BDI-II, PHQ-9, GAD-7, RSE, EUROHIS-QOL 8, WAI and the DAS. At discharge, we furthermore assess the HAQ, satisfaction with inpatient treatment, and acceptance of and satisfaction with deprexis® (intervention) or the online information (control condition). At the end of the intervention (T2) subjects fill out the BDI-II and a questionnaire concerning acceptance and satisfaction analogous to T1, including items assessing willingness to pay for the intervention (deprexis®). In addition, utilization of other treatments after the end of inpatient treatment will be measured at T3 (Table 1).

**Table 1:** **Schematic overview of frequency and scope of the study visits**

	**Screening**	**Pretreatment**	**Discharge**	**End of Intervention**	**Follow-up**
Study visits		T0	T1	T2	T3
Written informed consent	X				
Screening questions	X				
BDI-II	X		X	X	X
GAD-7		X	X		X
PHQ-9		X	X		X
RSE		X	X		X
EUROHIS-QOL 8		X	X		X
DAS		X	X		X
CTQ		X			
WAI		X	X		X
OPD-SQ		X			
HAQ			X		
Satisfaction with intervention			X	X	
Utilization of other treatment					X
BADO		X	X		
Willingness to pay				X	
Adverse events		continuously	X	X	

BADO, basic documentation; BDI-II, Beck Depression Inventory-II; CTQ, Childhood Trauma Questionnaire; DAS, Dysfunctional Attitude Scale; EUROHIS-QOL 8, European Health Interview Survey Quality of Life 8 Item Index; GAD, Generalized Anxiety Screener; HAQ, Helping Alliance Questionnaire; OPD-SQ, OPD Structure Questionnaire; PHQ, Patient Health Questionnaire; RSE, Rosenberg Self-Esteem Scale; WAI, Work Ability Index.

### Objectives and hypotheses

The purpose of this study is to determine the efficacy of an online self-help program (deprexis®) for enhancing standard inpatient psychotherapeutic treatment at the end of the intervention. We hypothesize that the online self-help group has a lower BDI-II score at the end of the intervention (primary outcome). We further assume that this effect will already be measurable at termination of inpatient treatment and will be maintained up to the 3-month follow-up (secondary outcomes).

### Outcomes

As the primary endpoint, we defined reduction of BDI-II score in the intervention group (treatment as usual + deprexis®) compared to the control group (treatment as usual + online information) at T2 (end of intervention).

Key secondary endpoint(s):

1. Reduction of BDI-II score at discharge
2. Reduction of BDI-II score at 3-month follow-up
3. Reduction of depression (PHQ-9) and anxiety (GAD-7)
4. Reduction of dysfunctional depression related cognitions (DAS)
5. Improved self-esteem (RSE)
6. Improved quality of life (EUROHIS-QOL)
7. Therapeutic alliance (patient and therapist rating, HAQ)
8. Acceptance and utilization of deprexis® and trial participation
9. Acceptance and utilization of the information used in the control condition and trial participation
10.  Satisfaction with inpatient treatment and willingness to pay
11.  Improved working ability
12.  Remission from depression

Predictors of outcome:

1. Recurrent depression (medical history of depression, previous inpatient treatment of depression)
2. Childhood trauma (CTQ)
3. Degree of structural deficits (OPD-SQ)
4. Utilization of other treatment after inpatient treatment

A history of emotional abuse as assessed with the CTQ has been linked to depression in several studies [31,32]. Structural deficits, especially personality disorders, may also constitute a risk for the manifestation or recurrence of depression [33]. Thus, childhood trauma and structural deficits have a moderating effect on the primary outcome.

### Sample size calculation

In order to determine treatment effects, effect sizes are computed. Effect sizes achieved by deprexis® have been in the range between d = 0.58 and d = 1.24 in various studies [8,21]. As we use deprexis® as an adjunct to inpatient psychotherapy, we assume the lowest effect achieved to date. Based on a conservative estimate of an effect size of d = 0.5, a power of 0.80 and an alpha error of 0.05, a sample of n = 64 patients per group is required (calculation by GPower vs. 3.1[34]). Based on a total sample of n = 240 patients, n = 120 in the intervention and in the control group, this sample size will still be reached even when the drop-out rate exceeds 40%. All statistical analyses are performed with SPSS Statistics 21 (IBM Deutschland GmbH, Ehningen, Germany).

### Randomization

The assignment of patients to the intervention and control groups is achieved by block-randomization at a ratio of 1:1. With the help of the computer software Research Randomizer [35], randomization will be conducted centrally by the Study Center of Mental Disorders as an independent institution.

### Statistical methods

For the primary outcome criteria, analysis of covariance will be used to compare BDI-II scores at the end of treatment between the intervention and control condition, with baseline scores of the BDI-II as covariates.

Intent-to-treat analyses with multiple imputations to replace missing data as well as completer analyses will be conducted.

For the secondary analyses, self-report questionnaires (BDI-II, PHQ-9, GAD-7, RSE, EUROHIS-QOL 8, DAS, WAI, HAQ) will also be analyzed by analysis of covariance All analyses will be conducted on a two-sided level of significance of 0.05. Descriptive statistics showing the measurements over time will be presented whenever appropriate. In order to define the percentage of remitted patients, the reliable change index [36] will be computed. Patients with a post-treatment depression score in BDI-II below the cut-off of 13 and a reliable change regarding the reliable change index will be considered as remitted. Additionally, serious adverse events and drop-outs will be analyzed descriptively.

## Discussion

Online self-help approaches provide promising means to deliver evidence-based care to a wide range of patients suffering from depression. Unguided self-help (that is, without therapist support) has the highest potential to do so at low cost. However, there is a strong relationship between the degree of support and the effectiveness of these programs, which requires additional resources (for example, therapist time). To date, little is known about the use of an online self-help program as an adjunct to inpatient face-to-face psychotherapy, where it could increase the effectiveness of treatment and bridge the gap between inpatient treatment and social and vocational reintegration. We therefore wish to answer the question if online self-help improves the efficacy of inpatient psychotherapy. We further determine if there are greater gains during inpatient treatment and if it helps to maintain treatment gains after treatment termination. With deprexis® we chose an intervention with an established efficacy. As a control condition we took care to provide relevant information pertaining to the causes, treatments and self-help options of depression. As we provide new additional information on a weekly basis, thorough study of this information will require considerable time, which is about equivalent to the time needed for participation in deprexis®.

Additional issues pertain to the feasibility of online self-help; for example, if patients accept it and continue its use after termination of inpatient treatment. Providing information is an adequate control condition, as it has been shown that German patients have a lack of knowledge regarding mental disease [37]. Knowledge is a prerequisite to change health behavior, which should promote recovery from disease.

In our study design, it did not seem feasible to keep therapists blind to the intervention that patients received, as this may interfere with an intensive psychotherapy process. In order to reduce potential bias (for example, therapist expectation), we used patient self-reported depression score as the main outcome. Inpatient treatment is psychodynamically oriented, whereas the online program constitutes a blend of modules from different psychotherapeutic, mostly cognitive-behavioral, traditions. We do not train therapists in the interventions delivered in the online program in order to keep it as an adjunct. In order to deal with the concern of therapists about potential effects of the online program participation on the inpatient treatment process, we assess helping alliance both from the perspective of the therapist and the patient at treatment termination in order to make sure that participation in the behaviorally oriented online program does not interfere with the therapeutic relationship which is considered to be of paramount importance for outcome in psychodynamic treatment [38]. We also ask therapists specifically about their experience. As we surmise that a substantial part of the sample will continue or enter new outpatient psychotherapy in the first months after inpatient treatment [39], we also assess and control this variable.

We chose a commercially available program which has been thoroughly tested and empirically validated. Costs of the online self-help program are being covered by the hospital for this study, as online interventions are currently not reimbursed by the German health insurances. In order to answer the question if this approach is feasible without external funding, we will ask patients if they would be willing to pay for the online self-help program and to what extent.

Overall, our trial will be one of the few trials [10,40] adding an online self-help program to standard treatment, with the aims to determine both the feasibility and effectiveness of supplementing inpatient psychotherapy of depressed patients by online self-help. We hope to answer the question if an additional online self-help program could improve remission rates after inpatient psychotherapeutic treatment. Furthermore, our study results could help to develop future concepts of inpatient psychotherapeutic treatment and aftercare interventions, which seem important against the background of high rates of diagnosed depressive disorders in the German population and the lack of adequate outpatient treatment options.

## Trial status

The first patients were enrolled to the study on 1 July 2014. Follow-up assessments for the last included patients are expected to be completed by April 2016.

## Abbreviations

BDI-II: Beck Depression Inventory-II
CTQ: Childhood Trauma Questionnaire
DAS: Dysfunctional Attitude Scale
EUROHIS-QOL 8: European Health Interview Survey Quality of Life 8 Item Index
GAD: Generalized Anxiety Screener
HAQ: Helping Alliance Questionnaire
ICD-10: International Statistical Classification of Diseases and Related Health Problems, 10th revision
OPD-SQ: OPD Structure Questionnaire
PHQ: Patient Health Questionnaire
RSE: Rosenberg Self-Esteem Scale
WAI: Work Ability Index
